# 1,3-Dimesitylimidazolidinium tetra­chloridogold(III) dichloro­methane solvate

**DOI:** 10.1107/S1600536808031115

**Published:** 2008-10-04

**Authors:** Tesfamariam K. Hagos, Stefan D. Nogai, Liliana Dobrzańska, Stephanie Cronje

**Affiliations:** a Department of Chemistry, University of Stellenbosch, Private Bag X1, Matieland, South Africa

## Abstract

The title ionic compound, (C_21_H_27_N_2_)[AuCl_4_]·CH_2_Cl_2_, was obtained from the reaction of 1,3-dimesitylimidazolidinium chloride with *t*-BuOK and a solution of AuCl_3_ in tetra­hydro­furan. In the crystal structure, numerous weak C—H⋯Cl hydrogen bonds form double layers parallel to (100), which are further stabilized by π–π inter­actions between mesitylene rings [centroid–centroid distance = 4.308 (4) Å], resulting in the formation of a three-dimensional supra­molecular assembly.

## Related literature

For related literature, see: Arduengo *et al.* (1995 ▶); da Costa *et al.* (2007 ▶); Adé *et al.* (2004 ▶); Asaji *et al.* (2004 ▶); Makotchenko *et al.* (2006 ▶); Brammer *et al.* (2001 ▶).
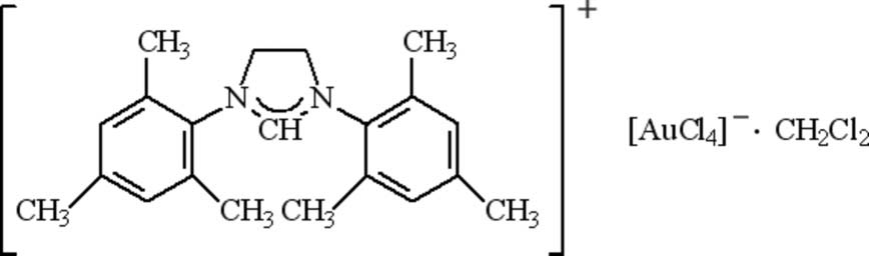


## Experimental

### 

#### Crystal data

(C_21_H_27_N_2_)[AuCl_4_]·CH_2_Cl_2_
*M* *_r_* = 731.14
Monoclinic, 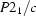

*a* = 19.590 (3) Å
*b* = 8.9986 (13) Å
*c* = 15.306 (2) Å
β = 96.601 (2)°
*V* = 2680.4 (7) Å^3^
*Z* = 4
Mo *K*α radiation
μ = 6.10 mm^−1^
*T* = 100 (2) K
0.30 × 0.25 × 0.10 mm

#### Data collection

Bruker APEX CCD area-detector diffractometer
Absorption correction: multi-scan (*SADABS*; Sheldrick, 1997 ▶) *T*_min_ = 0.146, *T*_max_ = 0.546
15546 measured reflections
6083 independent reflections
4516 reflections with *I* > 2σ(*I*)
*R*_int_ = 0.094

#### Refinement

*R*[*F* ^2^ > 2σ(*F* ^2^)] = 0.045
*wR*(*F* ^2^) = 0.086
*S* = 0.91
6083 reflections
286 parameters
H-atom parameters constrained
Δρ_max_ = 2.12 e Å^−3^
Δρ_min_ = −2.24 e Å^−3^

### 

Data collection: *SMART* (Bruker, 2001 ▶); cell refinement: *SAINT* (Bruker, 2002 ▶); data reduction: *SAINT*; program(s) used to solve structure: *SHELXS97* (Sheldrick, 2008 ▶); program(s) used to refine structure: *SHELXL97* (Sheldrick, 2008 ▶); molecular graphics: *X-SEED* (Barbour, 2001 ▶); software used to prepare material for publication: *X-SEED*.

## Supplementary Material

Crystal structure: contains datablocks I, global. DOI: 10.1107/S1600536808031115/hk2528sup1.cif

Structure factors: contains datablocks I. DOI: 10.1107/S1600536808031115/hk2528Isup2.hkl

Additional supplementary materials:  crystallographic information; 3D view; checkCIF report

## Figures and Tables

**Table 1:** Hydrogen-bond geometry (Å, °) (with cut-off parameters as in Brammer *et al*., 2001 ▶)

*D*—H⋯*A*	*D*—H	H⋯*A*	*D*⋯*A*	*D*—H⋯*A*
C9—H9⋯Cl1^i^	0.95	2.78	3.706 (6)	164
C12—H12*C*⋯Cl4^ii^	0.98	2.87	3.724 (6)	147
C15—H15⋯Cl3^ii^	0.95	2.87	3.740 (7)	152
C15—H15⋯Cl4^ii^	0.95	2.88	3.368 (6)	113
C17—H17*A*⋯Cl4	0.99	3.05	3.794 (6)	133
C17—H17*B*⋯Cl2^iii^	0.99	2.95	3.736 (6)	137
C17—H17*B*⋯Cl3^iii^	0.99	2.96	3.882 (6)	155
C18—H18*B*⋯Cl1	0.99	2.78	3.511 (6)	131
C25—H25*A*⋯Cl4^ii^	0.98	2.89	3.832 (6)	162
C25—H25*B*⋯Cl29^iv^	0.98	2.88	3.742 (7)	148
C25—H25*C*⋯Cl2^iii^	0.98	2.97	3.901 (6)	160
C25—H25*C*⋯Cl3^iii^	0.98	3.03	3.691 (6)	126
C26—H26*B*⋯Cl4	0.98	2.90	3.829 (7)	158
C27—H27*C*⋯Cl30^v^	0.98	3.04	3.838 (8)	140
C28—H28*A*⋯Cl3	0.99	2.63	3.486 (7)	145

Symmetry codes: (i) 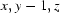; (ii) 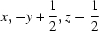; (iii) 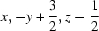; (iv) 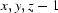; (v) 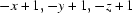.

**Fig. 1.**
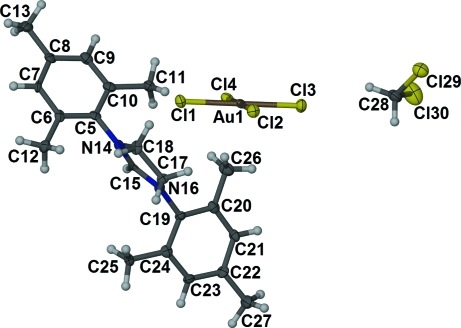
The molecular structure of the title compound, with the atom-numbering scheme. Displacement ellipsoids are drawn at the 50% probability level.

**Fig. 2.**
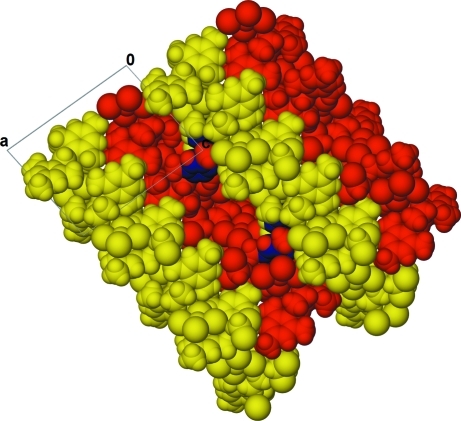
Space-filling representation of double layers (yellow-red) extended in the third dimension by π-π interactions (shown in blue).

## supplementary crystallographic information

### Comment

During the course of ongoing studies on imine compounds of gold(III), we have isolated the title ionic compound, (I). The asymmetric unit (Fig. 1) consists of 1,3-dimesitylimidazolidinium cation, tetrachloro-gold(III) anion, and a dichloromethane molecule. To the best of our knowledge, there have been only two reports on crystal structures containing the title carbenium ion, presenting the structure of the 1,3-dimesitylimidazolidinium chloride acetonitrile solvate, (II), (Arduengo et al., 1995) and the imidazolidinium salt of a ruthenium(III) complex, (III) (da Costa et al.,  2007), respectively. The structural parameters associated with the carbenium ion are similar to the reported ones. The only difference is the orientation of one of the mesitylene rings (C19-C24), which is almost perpendicular with respect to the plane of the imidazolidinium ring. The dihedral angle between those two planes is 89.5 (3)°, whereas in previous reports both the mesitylene rings were more or less twisted with respect to the plane of the imidazolidinium ring [66.0 (3)° and 75.1 (3)°] for (II) and 82.0 (3)° for (III). This corresponds with the orientation of the other mesitylene ring (C5-C10) [72.1 (3)°] described here.

The anionic part displays a typical square-planar geometry around Au and the Au-Cl distances compare well with previously reported values (Adé et al.,  2004; Asaji et al.,  2004; Makotchenko et al.,  2006). All Cl atoms participate in the formation of weak C-H···Cl hydrogen bonds (Table 1) forming double layers in the bc plane [individual layers are linked by C27-H27C···Cl30 bonds with C···Cl distance of 3.838 (8) Å] which are further extended in the third dimension by face-to-face π-π interactions between mesitylene rings (C5-C10) of neighbouring double layers [symmetry code: 2 - x, -y, 1 - z] with centroid-centroid distance of 4.308 (4) Å (Fig. 2).

### Experimental

For the preparation of the title compound, 1,3-dimesitylimidazolidinium chloride (0.04 g, 1.2 mmol) in THF (20 ml) was treated with t-BuOK (0.13 g, 1.2 mmol) at room temperature, and then filtered through Celite into a solution of AuCl_3_ (0.35 g, 1.2 mmol) in THF (20 ml). The solvent was removed under reduced pressure. Orange crystals suitable for single crystal X-ray analysis were obtained from a dichloromethane solution layered with hexane at 253 K.

### Refinement

H atoms were positioned geometrically, with C-H = 0.95, 0.99 and 0.98 Å for aromatic, methylene and methyl H, respectively, and constrained to ride on their parent atoms with U_iso_(H) = xU_eq_(C), where x = 1.5 for methyl H and x = 1.2 for all other H atoms.

### Crystal data

**Table:**

(C_21_H_27_N_2_)[AuCl_4_]·CH_2_Cl_2_	*F*(000) = 1424
*M**_r_* = 731.14	*D*_x_ = 1.812 Mg m^−^^3^
Monoclinic, *P*2_1_/*c*	Mo *K*α radiation, λ = 0.71073 Å
Hall symbol: -P 2ybc	Cell parameters from 3906 reflections
*a* = 19.590 (3) Å	θ = 2.5–27.6°
*b* = 8.9986 (13) Å	µ = 6.10 mm^−^^1^
*c* = 15.306 (2) Å	*T* = 100 K
β = 96.601 (2)°	Plate, orange
*V* = 2680.4 (7) Å^3^	0.30 × 0.25 × 0.10 mm
*Z* = 4	

### Data collection

**Table:**

Bruker APEX CCD area-detector diffractometer	6083 independent reflections
Radiation source: fine-focus sealed tube	4516 reflections with *I* > 2σ(*I*)
graphite	*R*_int_ = 0.094
ω scans	θ_max_ = 28.3°, θ_min_ = 2.1°
Absorption correction: multi-scan (SADABS; Sheldrick, 1997)	*h* = −21→25
*T*_min_ = 0.146, *T*_max_ = 0.546	*k* = −9→11
15546 measured reflections	*l* = −19→19

### Refinement

**Table:**

Refinement on *F*^2^	Primary atom site location: structure-invariant direct methods
Least-squares matrix: full	Secondary atom site location: difference Fourier map
*R*[*F*^2^ > 2σ(*F*^2^)] = 0.045	Hydrogen site location: inferred from neighbouring sites
*wR*(*F*^2^) = 0.087	H-atom parameters constrained
*S* = 0.91	*w* = 1/[σ^2^(*F*_o_^2^) + (0.0211*P*)^2^] where *P* = (*F*_o_^2^ + 2*F*_c_^2^)/3
6083 reflections	(Δ/σ)_max_ = 0.002
286 parameters	Δρ_max_ = 2.12 e Å^−^^3^
0 restraints	Δρ_min_ = −2.24 e Å^−^^3^

### Special details

**Table:**

Geometry. All e.s.d.'s (except the e.s.d. in the dihedral angle between two l.s. planes) are estimated using the full covariance matrix. The cell e.s.d.'s are taken into account individually in the estimation of e.s.d.'s in distances, angles and torsion angles; correlations between e.s.d.'s in cell parameters are only used when they are defined by crystal symmetry. An approximate (isotropic) treatment of cell e.s.d.'s is used for estimating e.s.d.'s involving l.s. planes.
Refinement. Refinement of *F*^2^ against ALL reflections. The weighted *R*-factor *wR* and goodness of fit *S* are based on *F*^2^, conventional *R*-factors *R* are based on *F*, with *F* set to zero for negative *F*^2^. The threshold expression of *F*^2^ > σ(*F*^2^) is used only for calculating *R*-factors(gt) *etc*. and is not relevant to the choice of reflections for refinement. *R*-factors based on *F*^2^ are statistically about twice as large as those based on *F*, and *R*- factors based on ALL data will be even larger.

### Fractional atomic coordinates and isotropic or equivalent isotropic displacement parameters (Å^2^)

**Table:**

	*x*	*y*	*z*	*U*_iso_*/*U*_eq_	
Au1	0.816928 (12)	0.61599 (3)	0.706473 (15)	0.01554 (8)	
Cl1	0.90506 (8)	0.57587 (17)	0.62476 (10)	0.0221 (4)	
Cl2	0.86227 (8)	0.84160 (17)	0.74770 (10)	0.0229 (4)	
Cl3	0.72796 (8)	0.65262 (18)	0.78862 (11)	0.0240 (4)	
Cl4	0.76938 (8)	0.39359 (17)	0.65952 (10)	0.0220 (3)	
C5	0.8618 (3)	0.1217 (7)	0.3991 (4)	0.0168 (13)	
C6	0.9177 (3)	0.1185 (7)	0.3509 (4)	0.0184 (13)	
C7	0.9615 (3)	−0.0040 (7)	0.3640 (4)	0.0179 (14)	
H7	0.9997	−0.0101	0.3311	0.022*	
C8	0.9517 (3)	−0.1156 (7)	0.4218 (4)	0.0223 (14)	
C9	0.8956 (3)	−0.1060 (7)	0.4701 (4)	0.0234 (15)	
H9	0.8881	−0.1829	0.5106	0.028*	
C10	0.8503 (3)	0.0132 (7)	0.4605 (4)	0.0222 (15)	
C11	0.7906 (3)	0.0228 (8)	0.5168 (4)	0.0259 (16)	
H11C	0.7899	0.1217	0.5434	0.039*	
H11A	0.7965	−0.0526	0.5632	0.039*	
H11B	0.7471	0.0055	0.4796	0.039*	
C12	0.9314 (3)	0.2390 (7)	0.2867 (4)	0.0228 (15)	
H12C	0.8938	0.2418	0.2386	0.034*	
H12A	0.9747	0.2184	0.2628	0.034*	
H12B	0.9345	0.3352	0.3169	0.034*	
C13	0.9994 (4)	−0.2463 (8)	0.4345 (4)	0.0299 (17)	
H13B	0.9829	−0.3258	0.3937	0.045*	
H13C	1.0010	−0.2824	0.4952	0.045*	
H13A	1.0456	−0.2161	0.4231	0.045*	
N14	0.8162 (3)	0.2491 (6)	0.3895 (3)	0.0171 (12)	
C15	0.7516 (3)	0.2431 (7)	0.3605 (4)	0.0161 (13)	
H15	0.7305	0.1576	0.3327	0.019*	
N16	0.7176 (3)	0.3665 (5)	0.3729 (3)	0.0168 (11)	
C17	0.7660 (3)	0.4782 (7)	0.4162 (4)	0.0170 (14)	
H17A	0.7505	0.5136	0.4719	0.020*	
H17B	0.7707	0.5644	0.3772	0.020*	
C18	0.8333 (3)	0.3929 (7)	0.4333 (4)	0.0210 (14)	
H18A	0.8707	0.4441	0.4069	0.025*	
H18B	0.8471	0.3795	0.4971	0.025*	
C19	0.6464 (3)	0.4010 (7)	0.3447 (4)	0.0165 (13)	
C20	0.5985 (3)	0.3755 (7)	0.4036 (4)	0.0207 (14)	
C21	0.5323 (3)	0.4244 (7)	0.3781 (4)	0.0191 (14)	
H21	0.4983	0.4097	0.4168	0.023*	
C22	0.5134 (3)	0.4949 (7)	0.2971 (4)	0.0209 (15)	
C23	0.5620 (3)	0.5118 (7)	0.2400 (4)	0.0210 (15)	
H23	0.5492	0.5574	0.1845	0.025*	
C24	0.6297 (3)	0.4639 (7)	0.2613 (4)	0.0202 (15)	
C25	0.6811 (3)	0.4777 (7)	0.1960 (4)	0.0230 (15)	
H25C	0.7222	0.5291	0.2236	0.034*	
H25B	0.6609	0.5347	0.1449	0.034*	
H25A	0.6938	0.3785	0.1771	0.034*	
C26	0.6168 (4)	0.2988 (8)	0.4893 (4)	0.0303 (17)	
H26C	0.5780	0.3048	0.5243	0.045*	
H26B	0.6571	0.3467	0.5212	0.045*	
H26A	0.6272	0.1942	0.4787	0.045*	
C27	0.4414 (3)	0.5523 (9)	0.2740 (5)	0.0317 (18)	
H27C	0.4317	0.5619	0.2100	0.048*	
H27A	0.4369	0.6496	0.3015	0.048*	
H27B	0.4087	0.4826	0.2955	0.048*	
C28	0.6210 (4)	0.7360 (8)	0.9489 (4)	0.0303 (17)	
H28B	0.6054	0.8168	0.9075	0.036*	
H28A	0.6592	0.6833	0.9251	0.036*	
Cl29	0.65120 (9)	0.8137 (2)	1.05262 (12)	0.0335 (4)	
Cl30	0.55384 (12)	0.6129 (3)	0.95642 (15)	0.0580 (6)	

### Atomic displacement parameters (Å^2^)

**Table:**

	*U*^11^	*U*^22^	*U*^33^	*U*^12^	*U*^13^	*U*^23^
Au1	0.01694 (12)	0.01813 (12)	0.01117 (12)	−0.00007 (12)	0.00005 (8)	0.00093 (11)
Cl1	0.0243 (8)	0.0229 (8)	0.0202 (8)	−0.0012 (7)	0.0073 (7)	−0.0006 (6)
Cl2	0.0252 (9)	0.0197 (8)	0.0236 (9)	−0.0021 (7)	0.0019 (7)	−0.0038 (6)
Cl3	0.0232 (8)	0.0241 (9)	0.0263 (9)	0.0015 (7)	0.0089 (7)	0.0007 (7)
Cl4	0.0268 (8)	0.0220 (8)	0.0167 (8)	−0.0060 (7)	0.0004 (6)	−0.0012 (7)
C5	0.019 (3)	0.019 (3)	0.010 (3)	−0.002 (3)	−0.004 (2)	−0.002 (3)
C6	0.014 (3)	0.027 (4)	0.014 (3)	−0.002 (3)	−0.002 (2)	−0.008 (3)
C7	0.011 (3)	0.028 (4)	0.015 (3)	0.004 (3)	0.002 (3)	−0.009 (3)
C8	0.032 (4)	0.024 (3)	0.010 (3)	0.007 (3)	−0.003 (3)	−0.004 (3)
C9	0.029 (4)	0.024 (4)	0.017 (3)	−0.003 (3)	0.001 (3)	−0.001 (3)
C10	0.024 (4)	0.027 (4)	0.015 (4)	0.000 (3)	−0.004 (3)	0.001 (3)
C11	0.027 (4)	0.031 (4)	0.020 (4)	0.006 (3)	0.006 (3)	0.007 (3)
C12	0.015 (3)	0.027 (4)	0.027 (4)	0.001 (3)	0.005 (3)	0.002 (3)
C13	0.036 (4)	0.039 (4)	0.014 (4)	0.013 (4)	−0.003 (3)	−0.003 (3)
N14	0.014 (3)	0.025 (3)	0.012 (3)	0.001 (2)	0.000 (2)	−0.002 (2)
C15	0.021 (3)	0.019 (3)	0.008 (3)	−0.005 (3)	0.002 (3)	0.006 (2)
N16	0.017 (3)	0.015 (3)	0.018 (3)	0.000 (2)	0.002 (2)	−0.004 (2)
C17	0.015 (3)	0.017 (3)	0.017 (3)	−0.002 (3)	−0.004 (3)	−0.003 (3)
C18	0.013 (3)	0.025 (4)	0.025 (3)	−0.007 (3)	0.002 (3)	−0.006 (3)
C19	0.018 (3)	0.014 (3)	0.017 (3)	0.003 (3)	0.001 (3)	0.001 (3)
C20	0.025 (3)	0.019 (3)	0.018 (3)	−0.003 (3)	0.003 (3)	−0.004 (3)
C21	0.017 (3)	0.024 (4)	0.017 (3)	−0.006 (3)	0.004 (3)	0.000 (3)
C22	0.011 (3)	0.025 (4)	0.026 (4)	−0.002 (3)	0.000 (3)	−0.001 (3)
C23	0.022 (4)	0.023 (4)	0.018 (4)	0.008 (3)	−0.002 (3)	0.000 (3)
C24	0.021 (4)	0.020 (3)	0.019 (4)	−0.004 (3)	0.001 (3)	−0.004 (3)
C25	0.026 (4)	0.028 (4)	0.016 (4)	0.003 (3)	0.006 (3)	−0.001 (3)
C26	0.030 (4)	0.035 (4)	0.026 (4)	−0.006 (3)	0.002 (3)	0.004 (3)
C27	0.020 (4)	0.049 (5)	0.026 (4)	0.006 (4)	0.005 (3)	0.006 (4)
C28	0.039 (4)	0.032 (4)	0.020 (4)	0.004 (4)	0.005 (3)	0.004 (3)
Cl29	0.0332 (10)	0.0352 (10)	0.0312 (10)	−0.0055 (9)	0.0001 (8)	0.0011 (8)
Cl30	0.0640 (15)	0.0613 (15)	0.0492 (13)	−0.0310 (13)	0.0087 (11)	−0.0151 (12)

### Geometric parameters (Å, °)

**Table:**

Au1—Cl1	2.2742 (16)	N16—C17	1.484 (7)
Au1—Cl2	2.2759 (16)	C17—C18	1.523 (8)
Au1—Cl3	2.2872 (16)	C17—H17A	0.9900
Au1—Cl4	2.2881 (16)	C17—H17B	0.9900
C5—C6	1.390 (8)	C18—H18A	0.9900
C5—C10	1.391 (8)	C18—H18B	0.9900
C5—N14	1.449 (8)	C19—C20	1.392 (9)
C6—C7	1.397 (8)	C19—C24	1.401 (8)
C6—C12	1.508 (8)	C20—C21	1.383 (9)
C7—C8	1.367 (9)	C20—C26	1.489 (9)
C7—H7	0.9500	C21—C22	1.404 (9)
C8—C9	1.397 (9)	C21—H21	0.9500
C8—C13	1.501 (9)	C22—C23	1.373 (9)
C9—C10	1.389 (9)	C22—C27	1.505 (8)
C9—H9	0.9500	C23—C24	1.397 (8)
C10—C11	1.533 (9)	C23—H23	0.9500
C11—H11C	0.9800	C24—C25	1.503 (9)
C11—H11A	0.9800	C25—H25C	0.9800
C11—H11B	0.9800	C25—H25B	0.9800
C12—H12C	0.9800	C25—H25A	0.9800
C12—H12A	0.9800	C26—H26C	0.9800
C12—H12B	0.9800	C26—H26B	0.9800
C13—H13B	0.9800	C26—H26A	0.9800
C13—H13C	0.9800	C27—H27C	0.9800
C13—H13A	0.9800	C27—H27A	0.9800
N14—C15	1.294 (7)	C27—H27B	0.9800
N14—C18	1.478 (8)	C28—Cl30	1.734 (7)
C15—N16	1.320 (8)	C28—Cl29	1.773 (7)
C15—H15	0.9500	C28—H28B	0.9900
N16—C19	1.446 (7)	C28—H28A	0.9900
			
Cl1—Au1—Cl2	89.85 (6)	C18—C17—H17A	111.1
Cl1—Au1—Cl3	179.15 (6)	N16—C17—H17B	111.1
Cl2—Au1—Cl3	90.96 (6)	C18—C17—H17B	111.1
Cl1—Au1—Cl4	89.76 (6)	H17A—C17—H17B	109.1
Cl2—Au1—Cl4	177.57 (6)	N14—C18—C17	102.5 (4)
Cl3—Au1—Cl4	89.44 (6)	N14—C18—H18A	111.3
C6—C5—C10	122.8 (6)	C17—C18—H18A	111.3
C6—C5—N14	118.4 (5)	N14—C18—H18B	111.3
C10—C5—N14	118.6 (6)	C17—C18—H18B	111.3
C5—C6—C7	116.7 (6)	H18A—C18—H18B	109.2
C5—C6—C12	122.7 (6)	C20—C19—C24	123.6 (6)
C7—C6—C12	120.6 (6)	C20—C19—N16	118.1 (5)
C8—C7—C6	122.9 (6)	C24—C19—N16	118.3 (5)
C8—C7—H7	118.6	C21—C20—C19	116.4 (6)
C6—C7—H7	118.6	C21—C20—C26	121.3 (6)
C7—C8—C9	118.4 (6)	C19—C20—C26	122.4 (6)
C7—C8—C13	121.9 (6)	C20—C21—C22	122.5 (6)
C9—C8—C13	119.7 (6)	C20—C21—H21	118.7
C10—C9—C8	121.5 (6)	C22—C21—H21	118.7
C10—C9—H9	119.2	C23—C22—C21	118.7 (6)
C8—C9—H9	119.2	C23—C22—C27	120.8 (6)
C9—C10—C5	117.7 (6)	C21—C22—C27	120.5 (6)
C9—C10—C11	120.2 (6)	C22—C23—C24	121.8 (6)
C5—C10—C11	122.2 (6)	C22—C23—H23	119.1
C10—C11—H11C	109.5	C24—C23—H23	119.1
C10—C11—H11A	109.5	C23—C24—C19	116.9 (6)
H11C—C11—H11A	109.5	C23—C24—C25	120.7 (6)
C10—C11—H11B	109.5	C19—C24—C25	122.4 (6)
H11C—C11—H11B	109.5	C24—C25—H25C	109.5
H11A—C11—H11B	109.5	C24—C25—H25B	109.5
C6—C12—H12C	109.5	H25C—C25—H25B	109.5
C6—C12—H12A	109.5	C24—C25—H25A	109.5
H12C—C12—H12A	109.5	H25C—C25—H25A	109.5
C6—C12—H12B	109.5	H25B—C25—H25A	109.5
H12C—C12—H12B	109.5	C20—C26—H26C	109.5
H12A—C12—H12B	109.5	C20—C26—H26B	109.5
C8—C13—H13B	109.5	H26C—C26—H26B	109.5
C8—C13—H13C	109.5	C20—C26—H26A	109.5
H13B—C13—H13C	109.5	H26C—C26—H26A	109.5
C8—C13—H13A	109.5	H26B—C26—H26A	109.5
H13B—C13—H13A	109.5	C22—C27—H27C	109.5
H13C—C13—H13A	109.5	C22—C27—H27A	109.5
C15—N14—C5	124.6 (5)	H27C—C27—H27A	109.5
C15—N14—C18	110.7 (5)	C22—C27—H27B	109.5
C5—N14—C18	122.8 (5)	H27C—C27—H27B	109.5
N14—C15—N16	113.9 (6)	H27A—C27—H27B	109.5
N14—C15—H15	123.0	Cl30—C28—Cl29	111.7 (4)
N16—C15—H15	123.0	Cl30—C28—H28B	109.3
C15—N16—C19	128.6 (5)	Cl29—C28—H28B	109.3
C15—N16—C17	109.1 (5)	Cl30—C28—H28A	109.3
C19—N16—C17	122.2 (5)	Cl29—C28—H28A	109.3
N16—C17—C18	103.4 (5)	H28B—C28—H28A	107.9
N16—C17—H17A	111.1		
			
C10—C5—C6—C7	2.7 (9)	C19—N16—C17—C18	−180.0 (5)
N14—C5—C6—C7	178.1 (5)	C15—N14—C18—C17	−5.3 (7)
C10—C5—C6—C12	−177.8 (6)	C5—N14—C18—C17	−170.6 (5)
N14—C5—C6—C12	−2.4 (8)	N16—C17—C18—N14	5.5 (6)
C5—C6—C7—C8	−1.1 (9)	C15—N16—C19—C20	94.1 (8)
C12—C6—C7—C8	179.4 (6)	C17—N16—C19—C20	−91.1 (7)
C6—C7—C8—C9	−0.2 (9)	C15—N16—C19—C24	−88.5 (8)
C6—C7—C8—C13	179.9 (6)	C17—N16—C19—C24	86.3 (7)
C7—C8—C9—C10	−0.1 (9)	C24—C19—C20—C21	−3.9 (9)
C13—C8—C9—C10	179.9 (6)	N16—C19—C20—C21	173.3 (5)
C8—C9—C10—C5	1.6 (9)	C24—C19—C20—C26	175.6 (6)
C8—C9—C10—C11	−177.9 (6)	N16—C19—C20—C26	−7.2 (9)
C6—C5—C10—C9	−3.0 (9)	C19—C20—C21—C22	0.6 (9)
N14—C5—C10—C9	−178.4 (5)	C26—C20—C21—C22	−178.9 (6)
C6—C5—C10—C11	176.6 (6)	C20—C21—C22—C23	2.0 (10)
N14—C5—C10—C11	1.1 (9)	C20—C21—C22—C27	−177.2 (6)
C6—C5—N14—C15	118.6 (7)	C21—C22—C23—C24	−1.4 (10)
C10—C5—N14—C15	−65.8 (8)	C27—C22—C23—C24	177.8 (6)
C6—C5—N14—C18	−78.2 (7)	C22—C23—C24—C19	−1.6 (9)
C10—C5—N14—C18	97.4 (7)	C22—C23—C24—C25	177.3 (6)
C5—N14—C15—N16	167.8 (5)	C20—C19—C24—C23	4.5 (9)
C18—N14—C15—N16	2.9 (7)	N16—C19—C24—C23	−172.8 (5)
N14—C15—N16—C19	176.4 (6)	C20—C19—C24—C25	−174.5 (6)
N14—C15—N16—C17	1.0 (7)	N16—C19—C24—C25	8.3 (9)
C15—N16—C17—C18	−4.3 (6)		

### Hydrogen-bond geometry (Å, °)

**Table:**

*D*—H···*A*	*D*—H	H···*A*	*D*···*A*	*D*—H···*A*
C9—H9···Cl1^i^	0.95	2.78	3.706 (6)	164
C12—H12C···Cl4^ii^	0.98	2.87	3.724 (6)	147
C15—H15···Cl3^ii^	0.95	2.87	3.740 (7)	152
C15—H15···Cl4^ii^	0.95	2.88	3.368 (6)	113
C17—H17A···Cl4	0.99	3.05	3.794 (6)	133
C17—H17B···Cl2^iii^	0.99	2.95	3.736 (6)	137
C17—H17B···Cl3^iii^	0.99	2.96	3.882 (6)	155
C18—H18B···Cl1	0.99	2.78	3.511 (6)	131
C25—H25A···Cl4^ii^	0.98	2.89	3.832 (6)	162
C25—H25B···Cl29^iv^	0.98	2.88	3.742 (7)	148
C25—H25C···Cl2^iii^	0.98	2.97	3.901 (6)	160
C25—H25C···Cl3^iii^	0.98	3.03	3.691 (6)	126
C26—H26B···Cl4	0.98	2.90	3.829 (7)	158
C27—H27C···Cl30^v^	0.98	3.04	3.838 (8)	140
C28—H28A···Cl3	0.99	2.63	3.486 (7)	145

Symmetry codes: (i) *x*, *y*−1, *z*; (ii) *x*, −*y*+1/2, *z*−1/2;  (iii) *x*, −*y*+3/2, *z*−1/2; (iv) *x*, *y*, *z*−1; (v) −*x*+1, −*y*+1, −*z*+1.
